# Emodin Attenuates Lipopolysaccharide-Induced Acute Liver Injury via Inhibiting the TLR4 Signaling Pathway *in vitro* and *in vivo*

**DOI:** 10.3389/fphar.2018.00962

**Published:** 2018-08-22

**Authors:** Yan Ding, Pan Liu, Zhi-Lin Chen, Shao-Jun Zhang, You-Qin Wang, Xin Cai, Lei Luo, Xuan Zhou, Lei Zhao

**Affiliations:** ^1^Department of Infectious Diseases and Immunology, Wuhan Children’s Hospital, Tongji Medical College, Huazhong University of Science and Technology, Wuhan, China; ^2^School of Clinical Medicine, Hubei University of Chinese Medicine, Wuhan, China; ^3^Department of Infectious Diseases, Union Hospital, Tongji Medical College, Huazhong University of Science and Technology, Wuhan, China; ^4^National and Local Joint Engineering Research Center for High-throughput Drug Screening Technology, Hubei Collaborative Innovation Center for Green Transformation of Bio-resources, Hubei University, Wuhan, China; ^5^Graduate School of Jinzhou Medical University, Department of Pediatrics, Renmin Hospital, Hubei University of Medicine, Shiyan, China

**Keywords:** emodin, lipopolysaccharide, TLR4, acute liver injury, signaling pathway

## Abstract

**Aims:** Emodin is an anthraquinone with potential anti-inflammatory properties. However, the possible molecular mechanisms and protective effects of emodin are not clear. The objective of this study was to investigate the possible molecular mechanisms and protective effects of emodin on lipopolysaccharide (LPS)-induced acute liver injury (ALI) via the Toll-like receptor 4 (TLR4) signaling pathway in the Raw264.7 cell line and in Balb/c mice.

**Methods:** This study established an inflammatory cellular model and induced an ALI animal model. TLR4 was overexpressed by lentivirus and downregulated by small interfering RNA (siRNA) technology. The mRNA and protein levels of TLR4 and downstream molecules were detected in cells and liver tissue. The tumor necrosis factor-α (TNF-α) and interleukin (IL)-6 levels in supernatant and serum were determined by ELISA. The distribution and expression of mannose receptor C type 1 (CD206) and arginase 1 (ARG1) in the liver were tested by immunofluorescence. Mouse liver function and histopathological observations were assessed.

**Results:** Administration of emodin reduced the protein and/or mRNA levels of TLR4 and its downstream molecules following LPS challenge in Raw264.7 cells and in an animal model. Additionally, emodin suppressed the expression of TNF-α and IL-6 in cell culture supernatant and serum. The inhibitory effect of emodin was also confirmed in RAW264.7 cells, in which TLR4 was overexpressed or knocked down. Additionally, ARG1 and CD206 were elevated in the emodin groups. Emodin also decreased serum ALT and AST levels and alleviated the liver histopathological damage induced by LPS.

**Conclusion:** Emodin showed excellent hepatoprotective effects against LPS-induced ALI, possibly by inhibiting TLR4 signaling pathways.

## Introduction

Hepatic disorder is a major disease endangering human health. Lipopolysaccharide (LPS)-induced liver injury belongs to a class of liver inflammatory response syndromes. This type of injury exhibits a high incidence, high fatality rate, high cost, and increasing morbidity (Liu et al., 2017). Inflammation is an early protective response to foreign pathogens and tissue injury. With early inflammatory activation, macrophages, a type of immunological cell, have an important role in both innate and adaptive immune systems (Sun et al., 2016; Zhu et al., 2016). LPS, a large molecule consisting of a polysaccharide and a lipid, is the main constituent of the outer membrane of intracellular Gram-negative bacteria and can activate liver macrophages, which advances the secretion of inflammatory cytokines. LPS damages the liver by serving as a hepatotoxin (Gao et al., 2015; Molinett et al., 2015; Tang et al., 2016; Singh et al., 2017). No specific treatment interventions are currently available for LPS-induced acute liver injury (ALI). Therefore, safe and efficacious treatment strategies against an LPS-induced ALI model are urgently needed (Chen et al., 2017).

The effects of LPS are mainly mediated via the membrane receptor TLR4 (Kumar et al., 2017). TLR4 is the chief TLR that mediates the Gram-negative LPS response (Kimoto et al., 2003; Döring et al., 2017; Kim et al., 2017). Unlike other Toll-like receptors (TLRs), the recognition of ligands by TLR4 requires the interaction of CD14 and myeloid differentiation 2 (MD-2) molecules (Triantafilou and Triantafilou, 2004; Resman et al., 2008; Hamesch et al., 2015), and TLR4 is localized and expressed on the cell surface and the endosomal membrane structure (Ma et al., 2018). LPS circulates into the liver through blood circulation, activating Kupffer cells and the TLR4 pathway (Wang et al., 2016; Singh et al., 2017). Then, LPS activates TLR4, which can initiate downstream signals via two main transduction signaling branches, myeloid differentiation factor88 (MyD88) and TIR-domain-containing adapter-inducing interferon-β (TRIF; Chattopadhyay et al., 2015; Wang, 2015). The TLR4 signaling pathway, via the two distinct branches of the MyD88- and TRIF-dependent pathways, activates the translocation of nuclear factor-kappa B (NF-κB) into the nucleus (Khan et al., 2016; Singh et al., 2017; Li et al., 2018). Both of these pathways play critical roles in the expression of a series of inflammatory factors, including tumor necrosis factor-α (TNF-α) and interleukin (IL)-6 (Ben et al., 2012; Hwang et al., 2017). In our study, we selected TLR4 as a putative signaling system underlying the LPS-induced ALI model for several reasons (Reino et al., 2011). On the one hand, activation of the TLR4-related signaling pathway has been implicated in the mechanism of organ injury, increased proinflammatory cytokine expression, and host defenses (Daubeuf et al., 2007; Avlas et al., 2011). TLR4 is absolutely necessary for triggering the innate response to LPS from Gram-negative bacteria. LPS activates liver macrophages, which advances the secretion of inflammatory cytokines (Guo et al., 2015a,b; Jiang et al., 2018). On the other hand, TLR4 has been widely demonstrated to be involved in the identification of pathogens, thereby implicating TLR4 as an innate immune sensor for bacterial infection and tissue injury (Mollen et al., 2006). Therefore, TLR4 activation might be an important pathway in the development of LPS-induced liver injury.

Rhubarb (Rheum rhabarbarum), a species of the perennial polygonaceae plant, is a well-known Chinese herbal medicine that has made great progress in clinical and pharmacological research. Emodin exerts protective effects on LPS-induced toxicity, which is consistent with a mechanism of action that is common to multiple classes of natural phenolic compounds (Menghini et al., 2016, 2018; Locatelli et al., 2018). All of these compounds have anti-inflammatory effects and are extracted from traditional herbs. The single rhubarb or rhubarb-based compound is commonly used in the treatment of various hepatobiliary diseases (Chen et al., 2015). In the treatment of liver diseases, emodin (1,3,8-trihydroxy-6-methyl-anthraquinone), as the main active component found in rhubarb, can alleviate liver injury by inhibiting inflammatory cytokines (Ding et al., 2008), decreasing hepatic oxidative stress (Liu et al., 2014), exhibiting immunosuppressive effects (Qiu et al., 2017), reducing hepatic damage (Meng et al., 2005; Zhao et al., 2018), reducing the neutrophil infiltration, and so on (Meng et al., 2010). Our previous work revealed that emodin can relieve the pathological changes in alpha-naphthylisothiocyanate (ANIT)-induced intrahepatic cholestasis in a rat model compared with a dexamethasone (DEX) group (Ding et al., 2008). The treatment of inflammation is accordingly important in current clinical practice (Li et al., 2017).

In this study, we propose the hypothesis that emodin can reduce macrophage activation and intrahepatic proinflammatory cytokines and improve LPS-induced ALI via the TLR4 signaling pathway. Based on this hypothesis, we carried out the following studies.

## Materials and Methods

### Reagents

Emodin (Catalog No: E7881) and LPS (from *Escherichia coli* 055:B5, Catalog No: L2880) were obtained from Sigma-Aldrich (St. Louis, MO, United States). A Cell Counting Kit-8 (CCK-8) kit was procured from Dojindo (Kumamoto, Japan). The SYBR^®^ Premix Ex Taq^TM^ kit (Catalog No: RR420A), PrimeScript^TM^ RT Master Mix kit (Catalog No: RR036A), and RNAiso Plus (Catalog No: SD1410) were obtained from TaKaRa Biotechnology (Dalian, China). DEX was purchased from Xinxiang Changle Company (Xinxiang, China). The TNF-α (Catalog No: E-EL-M0049c) and IL-6 (Catalog No: E-EL-M0044c) ELISA kits were obtained from Elabscience Biotechnology (Wuhan, China). Rabbit anti-mouse TLR4, TICAM1 (TRIF, Protocol Number: DF6289), AP-1 (Activator protein 1, Catalog No: AF6090), and NF-κB (Catalog No: AF5006) were provided from Affinity Biosciences (Cincinnati, OH, United States). Anti-mouse MyD88 (Catalog No: ab2064), anti-mouse TRAF6 (TNF receptor-associated factor 6, Catalog No: EP591Y), anti-mouse TRIAP (TIR-domain-containing adapter protein, Catalog No: EPR3509), and anti-mouse IRF-5 (Interferon regulatory factor 5, Catalog No: EPR17067) antibodies were obtained from Abcam Biotechnology (Cambridge, MA, United States). Anti-mouse IRF-3 antibody (Protocol Number: ET1612-14) was obtained from Huabio (Hanzhou, China). Fetal bovine serum (FBS) and DMEM/HIGH GLUCOSE medium were purchased from HyClone (Logan, UT, United States).

### Cell Culture and Cytotoxicity of Emodin

The RAW264.7 cell line was purchased from the China Center for Type Culture Collection (CCTCC). DMEM medium mixed with 10% FBS was used to culture the cells in an incubator at 37°C with saturated humidity and 5% CO2. The cytotoxicity of emodin in RAW264.7 cells was evaluated using the CCK-8 assay, which was carried out as described in our previous study (Yang et al., 2017). The cells were cultured in 96-well plates (5 × 10^3^) in 100 μl medium per well overnight. Then, emodin was added at different concentrations (3.75, 7.5, 15, 30, 60, 120, 240, and 480 μg/ml, dissolved in serum-free DMEM medium). After 24 h, 10 μl CCK-8 solution was added into each well for 1 h away from light. Then, the absorbance values of all the wells were measured on a microplate reader at 450 nm. The experiment was repeated three times independently.

### Cellular Model Establishment and Intervention

Lipopolysaccharide was used to establish the cellular inflammatory model (Hung et al., 2017; Liu et al., 2017). The dosage and timing of LPS for activating RAW264.7 cells were tested. After the cells were cultured in 6-well plates to 80% density, the cell supernatants were removed, and LPS, at a concentration of 1 μg/ml, was added to the wells (Li et al., 2017). After 0, 6, 12, 24, or 48 h, the cells were collected to detect the protein level of the TLR4 downstream signaling molecule TRAF-6 by western blotting to explore the timing of their activity. Meanwhile, dosage experiments were performed; after placing the cells in 6-well plates and culturing them to 80% density, the cell supernatants were removed, and LPS, at concentrations of 0, 0.5, 1, and 2 μg/ml, were added to the wells. After 24 h, the cells were harvested to detect the level of the TLR4 downstream signaling molecule TRAF-6 by western blotting to determine the optimal dosage of LPS. RAW264.7 cells were divided into a normal group, a model group, a DEX (0.5 μg/ml, diluted in PBS medium) group (Yang et al., 2018) and emodin (15, 30, and 60 μg/ml, dissolved in DMSO and then diluted in PBS medium to a final DMSO concentration less than 0.1%) groups. After placing the cells in 6-well plates overnight and culturing to 80% density, LPS (1 μg/ml) was added to the wells for 2 h, and then DXM and emodin were added as described above. The model group was treated with LPS (1 μg/ml) only. The normal group was cultured in DMEM medium without any treatment and served as a negative control. After 24 h, the cells were collected for quantitative real-time PCR and western blot analysis, and the supernatants were harvested for ELISA tests.

### Small Interfering (si)RNA-Mediated TLR4 Knock-Down in RAW264.7 Cells

Mouse TLR4 small interfering RNA (siRNA) (sense 5′-GAAAUGAGCUGGUAAAGAATT-3′, antisense 5′-UUCUUUACCAGCUCAUUUCTT-3′) was synthesized by Oligobio (Beijing, China). RAW264.7 cells were cultured into 6-well plates and transfected using 20 pmol/ml lipofectamine 2000 (Invivogen, San Diego, CA, United States) and 2.5 pmol/ml siRNA duplexes according to the manufacturer’s instructions. The medium was changed 6 h later after siRNA transfection; then, the cells continued to be cultivated for up to 48 h. At 24 h before harvest, the experimental group was treated with emodin or DEX.

### Lentiviral Vector-Mediated TLR4 Overexpression in RAW264.7 Cells

For the overexpression, TLR4 and negative control lentiviral vectors were synthesized by GeneChem (Shanghai, China). Based on the procedure described in our previous study (Yang et al., 2017), GV492-TLR4/NC-EGFP was transfected into the 293T cell line, and the viral supernatant [Titer (TU/ml:2E+8)] was harvested after 48 h. RAW264.7 cells were cultured into 6-well or 96-well plates and transfected by lentivirus vector at a multiplicity of infection (MOI) of 60, according to the manufacturer’s instructions. The medium was changed 6 h later, and the cells continued to be cultivated for 72 h. The lentivirus-treated cells were then treated with emodin or DEX for another 24 h.

### Animals

To observe the effect of the selective activation of TLR4 on mice with LPS-induced ALI, 30 4-week-old specific pathogen-free (SPF) Balb/c male mice (18–22 g) were obtained from the Hubei Provincial Center for Disease Control and Prevention (Wuhan, China). The number of animals was calculated by referring to a previous article (Charan and Kantharia, 2013). The mice were bred and maintained on a cycle of 12/12 h (light/darkness) with a normal diet and water ad libitum as some studies have described (Tsao et al., 2010; Recknagel et al., 2012). All experiments and animal care complied with the guidelines of internationally accepted principles and Huazhong University of Science and Technology (Guo et al., 2015a).

### Animal Grouping and Treatment

Thirty mice were randomly divided into six groups: the normal group, model group, emodin groups (20, 40, and 80 mg/kg/day; Ding et al., 2008), and DEX group (1.8 mg/kg/day; Yang et al., 2018). Before establishing the ALI model, emodin (20, 40, or 80 mg/kg/day; at a concentration of 1, 2, or 4 mg/ml) and DEX (1.8 mg/kg/d, at a concentration of 90 μg/ml) were intragastrically administered to the mice in corresponding groups for 30 min. The mice in the model and normal groups were administered normal saline (0.9% NaCl). Then, the mice were intraperitoneally injected with LPS (20 mg/kg), except the normal group (Li et al., 2014). Twelve hours later, the mice were sacrificed after being anesthetized with 10% chloral hydrate, and blood samples and liver tissue were taken from the mice in all groups; serum was immediately separated by centrifugation.

### Specimen Collection

The procedure was described in our previous study (Yang et al., 2017). Blood samples and liver tissue were taken from the mice in all groups. Blood was collected in an anticoagulant test tube. After centrifugation at 3200 g (4°C, 15 min), the serum was separated and stored at -20°C for further analysis. The liver was taken from the abdominal cavity using an aseptic technique. After washing with normal saline, a portion of liver tissue was fixed with 4% formaldehyde and embedded in paraffin. Hematoxylin and eosin (HE) staining was used to observe hepatic pathological changes. Another portion was stored at -80°C for subsequent extraction of RNA and protein.

### RNA Isolation and Real-Time PCR

The procedure was carried out as previously described (Jin et al., 2017). The total RNA was isolated from RAW264.7 cells or liver tissues using TRIzol reagent (Takara, Dalian, China). Subsequently, reverse transcription (RNA to cDNA, incubated at 37°C/15 min, at 85°C/5 s and 4°C/pause.) was performed with Primescript RT Master Mix reagent according to the manual specifications. For real-time PCR, the amplification steps and the reaction conditions were selected according to the instructions (SYBR Premix Ex Taq kit, TAKARA). Thermal cycling conditions were 30 s at 95°C, 5 s at 95°C, and 30 s at 60°C, followed by 40 cycles at 95°C for 15 s, 60°C for 1 min, and 95°C for 15 s in StepOnePlus (Applied Biosystems). The mRNA expression results were analyzed according to the 2^-∆ ∆ CT^ method. All procedures were performed in triplicate. All primers were designed by PrimerBank verifying data obtained from the NCBI/BLAST and synthesized by GenScript (Nanjing, China). Primers for real-time PCR were as follows:

    Gene        primer sequence (5′→3′)

TLR4 forward: TCTGGGGAGGCACATCTTCT
  Reverse: AGGTCCAAGTTGCCGTTTCT
TRIF forward: GCAGAGTCGGGGTAACAAGA
  Reverse: CCAGAAGGTGGTGCTCAAATA
Myd88 forward: TCATGTTCTCCATACCCTTGGT
  Reverse: AAACTGCGAGTGGGGTCAG
TRIAP forward: CCTCCTCCACTCCGTCCAA
  Reverse: CTTTCCTGGGAGATCGGCAT
TRAf-6 forward: AAAGCGAGAGATTCTTTCCCTG
  Reverse: ACTGGGGACAATTCACTAGAGC
IRF-5 forward: GGTCAACGGGGAAAAGAAACT
  Reverse: CATCCACCCCTTCAGTGTACT
IRF-3 forward: CACTCTGTGGTTCTGCATGG
  Reverse: ATGTCCTCCACCAAGTCCTG
NF-κB forward: CGCAAGCCCTTCAGTGACATC
  Reverse: GGTACTGGCTGTCAGGGTGGTT
AP-1 forward: CCTTCTACGACGATGCCCTC
  Reverse: GGTTCAAGGTCATGCTCTGTTT
GAPDH forward: AGGTCGGTGTGAACGGATTTG
  Reverse: TGTAGACCATGTAGTTGAGGTCA

### Western Blotting Analysis

The western blotting technique followed the procedures of previous studies (Wang et al., 2017; Jiang et al., 2018). Proteins extracted from either liver tissue or cultured RAW264.7 cells were analyzed by western blotting. The protein concentration of each sample was determined by a BCA assay kit (Beyotime, Shanghai). Equal volumes of protein were added to each well and separated by 10% SDS-PAGE and were then transferred to a PVDF (0.45 μm) membrane. The membranes were blocked for 1 h in TBST containing Tween 20 (0.1%) and fat-free milk (5%) at 37°C. Subsequently, the membranes were washed with TBST buffer three times and placed in rabbit-anti mouse specific antibody solution and incubated at 4°C overnight. After washing three times, the membranes were incubated with secondary antibody for 1 h at room temperature. The membranes were further washed, and protein bands were identified by an enhanced chemiluminescence (ECL) reagent kit. The signal was detected using Fuji ultrasonic-Doppler velocity profile (UVP) system by exposing the membranes to an X-ray film for 5 min.

### Enzyme-Linked Immunosorbent Assay (ELISA)

The procedure was performed according to past experiments (Li et al., 2017; Jiang et al., 2018). The expression levels of TNF-α and IL-6 in the cell supernatants and mouse serum were determined by the ELISA method. The sensitivities of the mouse TNF-α and IL-6 ELISA kits were both 18.75 pg/mL, and the intra- and inter-assay coefficients of variation were both below 10%. The related ELISAs used in this study had no significant cross reaction with other related proteins. All samples were assayed in duplicate.

### Biochemical Tests

The levels of aspartate aminotransferase (AST) and alanine aminotransferase (ALT) in serum were measured by Aeroset fully automated Chemistry Analyzer provided by Abbott Co., Ltd. (Ding et al., 2008).

### Immunofluorescence (IF)

The slides were washed three times with PBS and blocked with 10% normal goat serum at room temperature for 30 min. Then, the slides were incubated with a primary antibody (CD206 1:100, ARG1 1:50) at 4°C overnight. The slides were washed with PBS and exposed to fluorescent secondary antibodies Cy3-conjugated goat anti-rabbit IgG (diluted 1:100, Boster, Wuhan) and FITC-conjugated goat anti-rabbit IgG (diluted 1:100, Boster, Wuhan) at 37°C for half an hour without light. Subsequently, after further washing in PBS, the slides were incubated with DAPI for 10 min and mounted.

### Statistical Analyses

All the data were analyzed with SPSS 12.0 software (Ding et al., 2015). Quantitative data were expressed as the mean ± SD. Student’s t-test was used to analyze the differences between two groups. Data were compared between multiple groups by ANOVA and the SNK test. The level of statistical significance was considered at *P* < 0.05 or *P* < 0.01. Figures were generated by GraphPad 5.0.

## Results

### *In vitro* Cytotoxicity of Emodin

**FIGURE 1:**
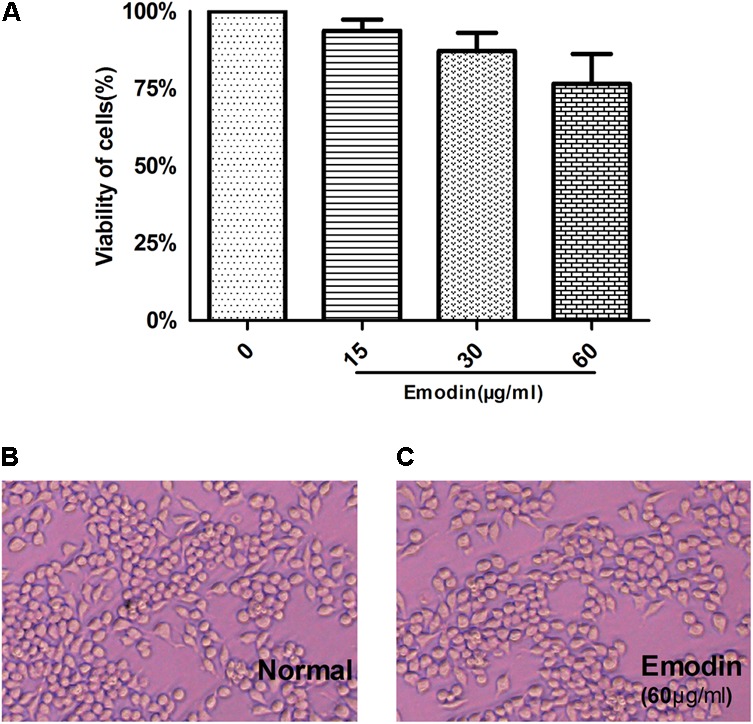
*In vitro* cytotoxicity of emodin. The cytotoxicity of emodin was evaluated by the CCK8 assay. **(A)** A CCK-8 assay of RAW264.7 cell viability after emodin treatment. **(B)** Morphologies of Raw264.7 cells. **(C)** Morphologies of Raw264.7 cells after emodin treatment for 24 h. Data are shown as the mean ± SD. *N* = 3.

*In vitro*, we assessed the viability of RAW264.7 cells pretreated with emodin at different concentrations (3.75, 7.5, 15, 30, 60, 120, 240, and 480 μg/ml) after 24 h. Based on the CCK-8 assay, the 75% viability of emodin concentration was calculated as 65.07 μg/ml. For convenience, we approximated high concentration as 60 μg/ml, middle concentration as 30 μg/ml, and low concentration as 15 μg/ml (Yang et al., 2018). As shown in **Figure 1A**, the CCK-8 assay showed the viability of RAW264.7 cells. Therefore, RAW 264.7 cells were treated with emodin at concentrations of 60, 30, and 15 μg/ml for 24 h. Furthermore, according to the cell morphological assay, the changes in cell morphological observation among the 60 μg/ml emodin group and the normal group were not significant (**Figures 1B,C**).

### Model Establishment

**FIGURE 2:**
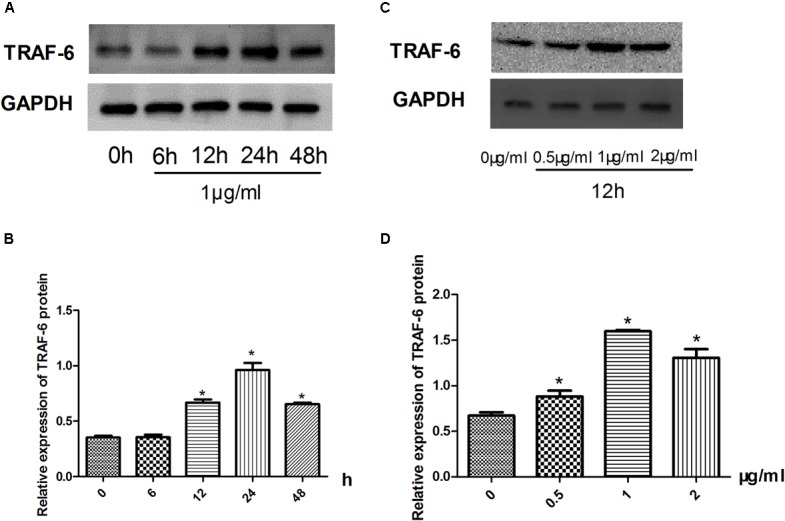
Inflammatory model establishment. **(A–D)** Effect of LPS stimulation on the expression of TRAF-6 according to western blotting. Data are shown as the mean ± SD. ^∗^*P* < 0.05 vs either time point 0 or LPS untreated group.

There was a dose- and time-dependent effect on the protein expression of TRAF-6 in Raw264.7 cells treated with LPS (**Figure 2**). The protein expression of TRAF-6 was measured after LPS stimulation at 1 μg/ml for 0–48 h (**Figure 2A**) and at a range of 0–2 μg/ml for 24 h in Raw264.7 cells (**Figure 2B**). As shown in **Figure 2C**, the protein expression of TRAF-6 reached its highest level at 24 h compared with the other groups. As shown in **Figure 2D**, with LPS stimulation at 1 μg/ml, the level of TRAF-6 increased significantly compared to other groups. Therefore, we chose LPS treatment at 1 μg/ml for 24 h as the optimal condition of LPS to activate the RAW 264.7 macrophage inflammatory model.

### Evaluation of TNF-α and IL-6 Expression in Cell Supernatant by ELISA

**FIGURE 3:**
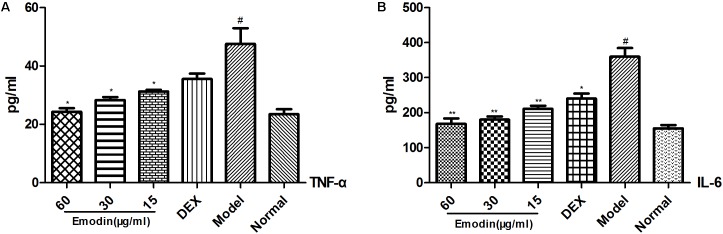
Evaluation of TNF-α and IL-6 expression in cell supernatants by ELISA. **(A,B)** Effect of emodin on the expression of TNF-α and IL-6 according to ELISA. Data shown are the mean ± SD. ^#^*P* < 0.05 vs normal group; ^∗^*P* < 0.05, ^∗∗^*P* < 0.01 vs model group.

As shown in **Figures 3A,B**, the levels of TNF-α and IL-6 in the cell supernatants significantly increased in the model group compared to the normal group (*P* < 0.05). The levels of TNF-α and IL-6 were markedly decreased compared with that in the model group when cells were treated with emodin at different doses (*P* < 0.05 or *P* < 0.01). The inhibitory effect was enhanced when the dose of emodin increased (*P* < 0.01), which indicated that emodin could suppress the expression of TNF-α and IL-6.

### Effect of Emodin on TLR4 and Downstream Molecules After LPS Stimulation

**FIGURE 4:**
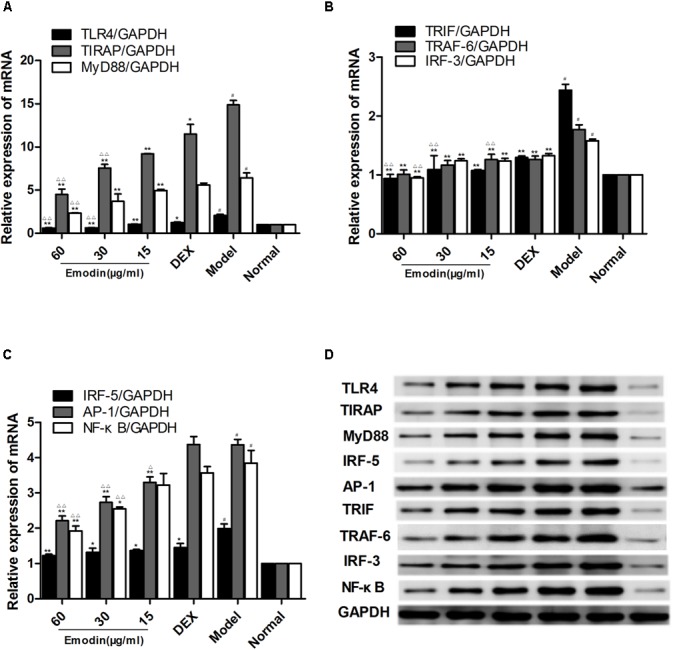
Effect of emodin on TLR4 and downstream molecules after LPS stimulation. **(A–C)** The mRNA levels of TLR4, MyD88, TIRAP, IRF-5, TRAF-6, TRIF, IRF-3, AP-1, and NF–κB were detected by RT-PCR. **(D)** The protein levels of the above molecules were detected by western blotting. Data are shown as the mean ± SD. ^#^*P* < 0.05 compared to the normal group. ^∗^*P* < 0.05, ^∗∗^*P* < 0.01 vs model group; ^∆^*P* < 0.05, ^∆ ∆^*P* < 0.01 vs DEX group.

As shown in **Figures 4A–D**, compared with the normal group, the mRNA and protein levels of TLR4 in the model group were markedly elevated. Meanwhile, LPS and emodin had opposite effects on the expression of TLR4. Moreover, the mRNA and protein expression levels of the downstream molecules MyD88, TIRAP, IRF-5, TRAF-6, TRIF, IRF-3, AP-1, and NF-κB were also markedly increased in the model group (*P* < 0.05). After treatment with different concentrations of emodin and DEX for 24 h, the expression of TLR4 was significantly decreased compared with the model group. More importantly, the decreases in the mRNA levels of the downstream molecules MyD88, TIRAP, TRIF, IRF-3, AP-1, and NF-κB in the emodin (60 μg/ml) group were more significantly declined than those in the DEX group (*P* < 0.01; **Figures 4A–C**); the protein levels of MyD88, TIRAP, TRIF, IRF-3, IRF-5, TRAF-6, AP-1, and NF-κB were also significantly lower (**Figure 4D**). Emodin treatment significantly reduced TLR4 and downstream molecule expression after LPS stimulation.

### Effect of Emodin on TLR4 and Downstream Molecules After TLR4 Knock-Down

**FIGURE 5:**
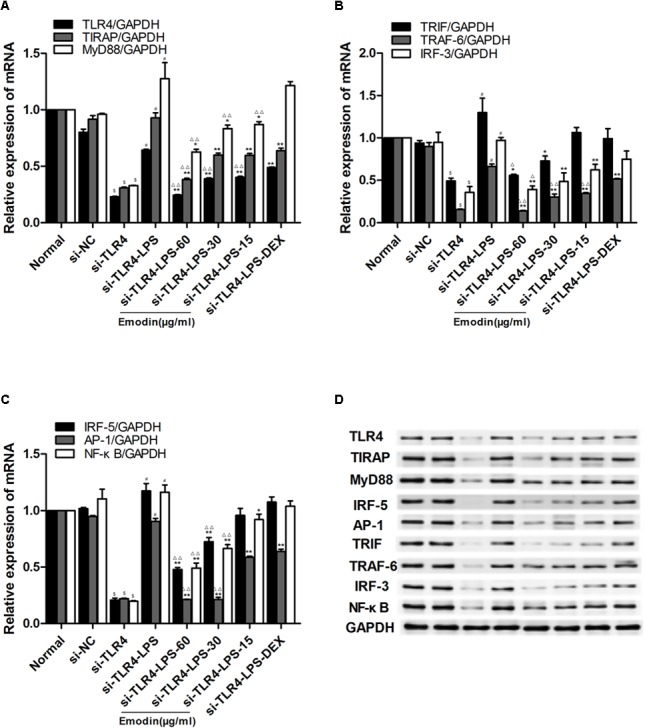
Effect of emodin on TLR4 and downstream molecules after TLR4 knock-down. Expression of TLR4 was observed with a fluorescence microscope after siRNA was introduced into cells for 48 h, and then the cells were treated with different drug intervention for 24 h. **(A–C)** The levels of TLR4 and downstream molecules MyD88, TIRAP, IRF-5, TRAF-6, TRIF, IRF-3, AP-1, and NF-κB after transfection were confirmed by real-time quantitative PCR. **(D)** Expression of protein was assayed by western blotting. Data are shown as the mean ± SD. ^$^*P* < 0.05 compared with the normal group; ^#^*P* < 0.05 compared with the Si-TLR4 group; ^∗^*P* < 0.05, ^∗∗^*P* < 0.01, compared with the Si-TLR4-LPS group; ^∆^*P* < 0.05, ^∆ ∆^*P* < 0.01, compared with the DEX group. si-TLR4: TLR4 was knocked down in Raw264.7 cells by siRNA. si-NC: SiRNA negative control group.

To precisely downregulate the expression of TLR4, we used siRNA to interfere with TLR4 in RAW264.7 cells. TLR4 was downregulated by siRNA in Raw264.7 cells for 48 h. The emodin groups, DEX group, and model group were stimulated by LPS (1 μg/ml) for 2 h and then emodin (60, 30, or 15 μg/ml), DEX was added. As shown in **Figures 5A–D**, we observed that there was no difference between the normal group and the si-NC group (siRNA negative control group; *P* > 0.05). The mRNA and protein levels of TLR4, MyD88, TIRAP, IRF-5, TRAF-6, TRIF, IRF-3, AP-1, and NF-κB in the si-TLR4 group were significantly decreased compared with the normal group (*P* < 0.01). Compared with the si-TLR4 group, the levels of TLR4 and its downstream molecules MyD88, TIRAP, IRF-5, TRAF-6, TRIF, IRF-3, AP-1, and NF-κB in the si-TLR4-LPS group were increased (*P* < 0.01). However, compared with the si-TLR4-LPS group, the emodin and DEX groups showed significantly inhibited mRNA expression levels of TLR4, TIRAP, TRAF-6, AP-1, and NF-κB (**Figures 5A–C**), as well as significantly reduced protein expression levels of TLR4, MyD88, TIRAP, TRAF-6, TRIF, IRF-3, and AP-1 (*P* < 0.05 or *P* < 0.01; **Figure 5D**). In addition, the TLR4, MyD88, IRF-5, TRAF-6, AP-1, and NF-κB mRNA and protein levels in the emodin (30, 60 μg/ml) groups, as well as the TIRAP, TRIF, and IRF-3 mRNA and protein levels in the emodin (60 μg/ml) group, were notably more reduced than those in the DEX group (*P* < 0.05 or *P* < 0.01).

### Effect of Emodin on TLR4 and Downstream Molecules After TLR4 Overexpression

**FIGURE 6:**
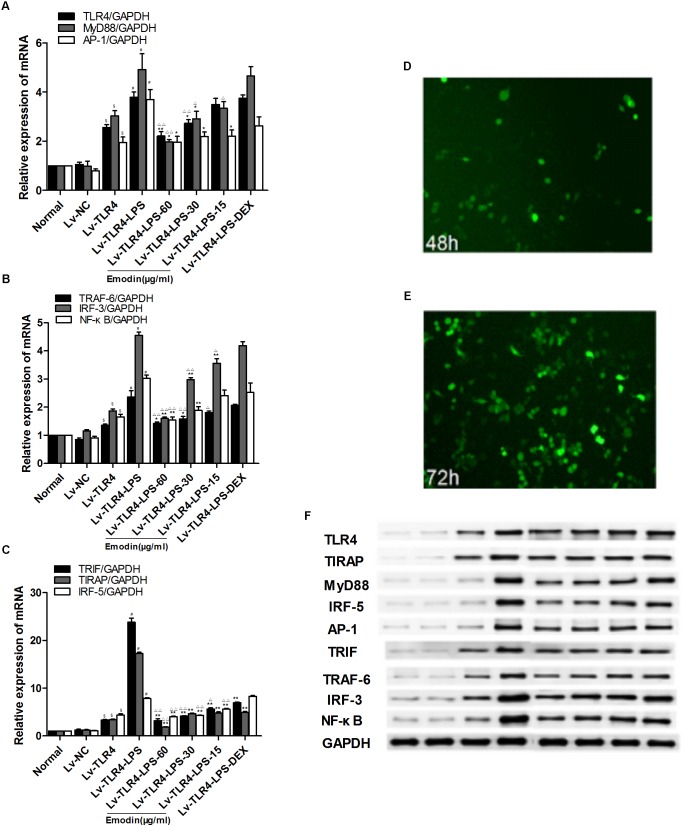
Effect of emodin on TLR4 and downstream molecules after TLR4 overexpression. Overexpression of TLR4 in Raw264.7 cells via the lentiviral vector. Lentivirus vector interferes with Raw264.7 cells for 72 h to mediate TLR4 overexpression. **(A–C)** The levels of TLR4 and downstream molecules MyD88, TIRAP, IRF-5, TRAF-6, TRIF, IRF-3, AP-1, and NF-κB after transfection were confirmed by real-time quantitative PCR. **(D,E)** The expression of TLR4 was observed with a fluorescence microscope after lentivirus was introduced into Raw264.7 cells for 48 and 72 h. **(F)** Expression of protein was assayed by western blotting. Data are shown as the mean ± SD. ^$^*P* < 0.05 compared with the normal group; ^#^*P* < 0.05 compared with the Lv-TLR4 group; ^∗∗^*P* < 0.05, ^∗^*P* < 0.01, compared with the Lv-TLR4-LPS group. ^∆^*P* < 0.05, ^∆ ∆^*P* < 0.01, compared with the DEX group. Lv-TLR4: TLR4 was overexpressed in Raw264.7 cells by lentivirus vector. Lv-NC: lentivirus vector negative control group.

We generated lentivirus-mediated TLR4 overexpression in RAW264.7 cells, and the expression of TLR4 was confirmed after 72 h. Green fluorescent protein (FEM) was observed with a fluorescence microscope; FEM fluorescent signal was detected after the lentivirus was transfected to cells for 48 and 72 h (**Figures 6D,E**). RAW264.7 cells were interfered with lentiviral vector for 72 h to mediate TLR4 overexpression. Then, LPS (1 μg/ml) was added for 2 h, except in the normal group; after that, emodin (60, 30, or 15 μg/ml) or DEX was added for 24 h. As shown in **Figures 6A–C, F,** we found that the mRNA and protein levels of TLR4 and downstream molecules were not different in the normal control group when compared with that in Lv-NC group (lentiviral vector negative control group; *P* > 0.05). The Lv-TLR4-LPS group showed significantly increased mRNA expression levels of TLR4, MyD88, TIRAP, IRF-5, TRAF-6, TRIF, IRF-3, AP-1, and NF-κB when compared with the Lv-TLR4 group (*P* < 0.01; **Figures 6A–C**), and the protein expression levels of those molecules were also significantly elevated (**Figure 6F**). The emodin and DEX groups demonstrated inhibited levels of TLR4 and downstream molecules. In addition, the TLR4, MyD88, IRF-5, TRAF-6, IRF-3, TRIF, TIRAP, and NF-κB mRNA levels in the emodin (60 μg/ml) group were notably more declined than those in the DEX group (*P* < 0.05 or *P* < 0.01). The protein expression of TLR4 and downstream molecules in the emodin-treated groups were significantly reduced when compared with those of the DEX group (*P* < 0.05 or *P* < 0.01). Therefore, we found that emodin might inhibit downstream molecules in RAW264.7 cells when TLR4 is overexpressed.

### Effect of Emodin on Serum TNF-α and IL-6

**FIGURE 7:**
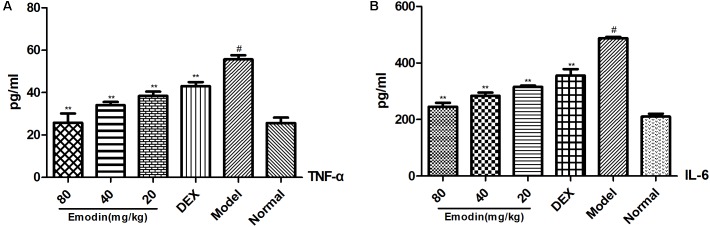
Effect of emodin on the expression of TNF-α and IL-6 in mouse serum. Effect of **(A,B)** emodin on the expression of TNF-α, IL-6 in mice serum was measured by ELISA. Data are shown as the mean ± SD. ^#^*P* < 0.05 vs normal group; ^∗^*P* < 0.05, ^∗∗^*P* < 0.01 vs model group.

The expression of serum TNF-α and IL-6 was tested with ELISA. As shown in **Figures 7A,B**, serum TNF-α and IL-6 levels in the model group were significantly increased when compared with those of the normal group (*P* < 0.01). TNF-α and IL-6 expression was significantly reduced compared with the model group with the emodin intervention (*P* < 0.01). Emodin had a remarkable effect on inhibiting the TNF-α and IL-6 levels (*P* < 0.05 or *P* < 0.01). DEX had a similar effect to emodin on TNF-α and IL-6 levels.

### Effect of Emodin on the TLR4 Signaling Pathway in a Mouse Model

**FIGURE 8:**
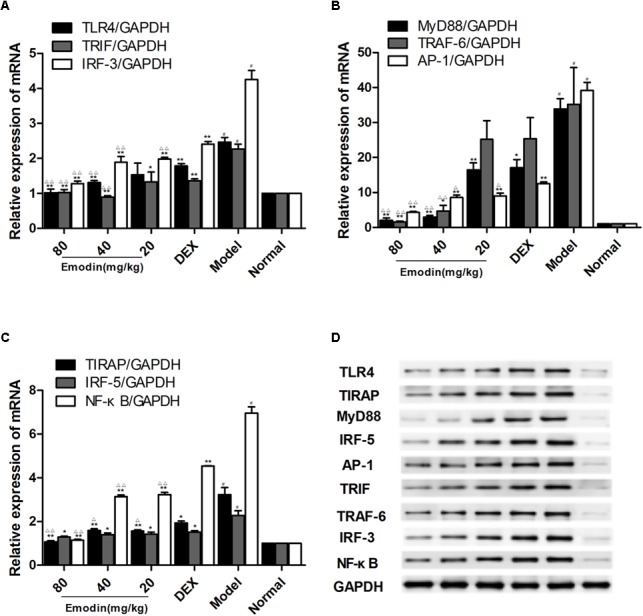
Effects of emodin on TLR4 signaling pathways in a mouse model. Effect of emodin on the expression of TLR4 and downstream molecule expression in a mouse model. **(A–C)** The mRNA levels of TLR4, MyD88, TIRAP, IRF-5, TRAF-6, TRIF, IRF-3, AP-1, and NF-κB were detected by RT-PCR. **(D)** The protein levels of above molecules were detected by western blotting. Data are shown as the mean ± SD. ^#^*P* < 0.05 compared with the normal group. ^∗^*P* < 0.05, ^∗∗^*P* < 0.01 vs model group; ^∆^*P* < 0.05, ^∆ ∆^*P* < 0.01, compared with the DEX group.

As shown in **Figures 8A–D**, compared with the normal group, the protein and/or mRNA levels of TLR4, MyD88, TIRAP, IRF-5, TRAF-6, TRIF, IRF-3, AP-1, and NF–κB were significantly increased in the model group (*P* < 0.01). The levels of TLR4 and downstream molecules in the emodin and DEX groups were decreased compared with those of the model group. In addition, the TLR4, MyD88, TLR4, MyD88, TRAF-6, IRF-3, TRIF, TIRAP, AP-1, and NF-κB mRNA levels in the 80 mg/kg emodin group (**Figures 8A–C**) and the TLR4, MyD88, IRF-5, TRAF-6, IRF-3, TRIF, TIRAP, AP-1, and NF-κB protein levels in the 80 and 40 mg/kg emodin groups were significantly lower than those in the DEX group (**Figure 8D**).

### Effect of Emodin on Liver Morphology

**FIGURE 9:**
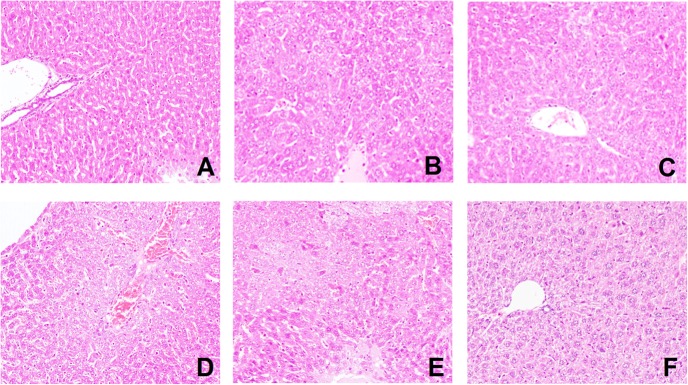
Effect of emodin on liver morphology. **(A–F)** Pathological changes were observed in the liver tissues with H&E staining (original magnification×100). The **(A)** emodin (80 mg/kg) group, **(B)** emodin (40 mg/kg) group, **(C)** emodin (20 mg/kg) group, **(D)** DEX group, **(E)** model group, and **(F)** normal group. In the liver, interstitial cells showed obvious edema. **(A–C)** Liver tissues from mice treated with different concentrations of emodin groups looked healthier compared with those from model mice infected with LPS.

The tissues obtained from the livers were further evaluated by H&E staining. As shown in **Figure 9**, pathological changes were observed in the liver tissues. As shown in **Figure 9F**, the liver tissue from the normal group exhibited normal hepatocytes and intact hepatic tissue architecture. The model group showed destruction of liver architecture and inflammatory cell infiltration (**Figure 9E**). Liver tissue from mice treated with different concentrations of emodin showed less prominent liver injury and less hepatic inflammatory cell infiltration (**Figures 9A–C**). The severe histological abnormalities had no significant changes in the DEX group (**Figure 9D**). These results suggest that emodin protects the liver from injury induced by LPS and thereby enhances cell viability.

### Effect of Emodin on Serum Biochemical Indicators

To clarify the effect of emodin on ALI, the activities of ALT and AST in serum were detected to evaluate liver dysfunction and hepatocellular injury (Chen et al., 2017). As shown in **Table 1**, compared with the normal group, LPS stimulation caused acute liver dysfunction in the model group as evidenced by a significant increase in serum liver enzyme activities. Compared with the model group, the emodin-treated groups showed significant decreases in ALT and AST levels (*n* = 5; *P* < 0.05). Emodin (80, 40, and 20 mg/kg) induced similar effects as DEX on ALT (*n* = 5; *P* < 0.05). The emodin-treated groups exhibited the greatest effects on AST, and the DEX group had non-significant efficacy on AST (*n* = 5; *P* > 0.05).

**Table 1:** Effect of emodin on serum biochemical indicators.

Group	ALT(U/l)	AST(U/l)
Emodin(80 mg/kg)	89.6 ± 3.5^#∗∗$^	260.4 ± 11.8^#∗∗^
Emodin(40 mg/kg)	95.8 ± 4.9^#∗∗$^	255.4 ± 21.4^#∗∗^
Emodin(20 mg/kg)	127.2 ± 11.2^#∗^	288.2 ± 22.2^#∗^
Dexamethasone	133.2 ± 12.1^#∗^	319.6 ± 28.8^#^
Model	199.6 ± 25.8^#^	363.8 ± 17.9^#^
Normal	36.6 ± 4.4	98.60 ± 5.7

*Effect of emodin on liver function tests. Data are shown as the mean ± SD. n = 5. ^#^P < 0.01 vs normal group; ^∗^P < 0.05, ^∗∗^P < 0.01 vs model group; ^$^P < 0.05 vs DEX group.*

### Effect of Emodin on M2 Macrophages in the Liver Measured by Immunofluorescence

**FIGURE 10:**
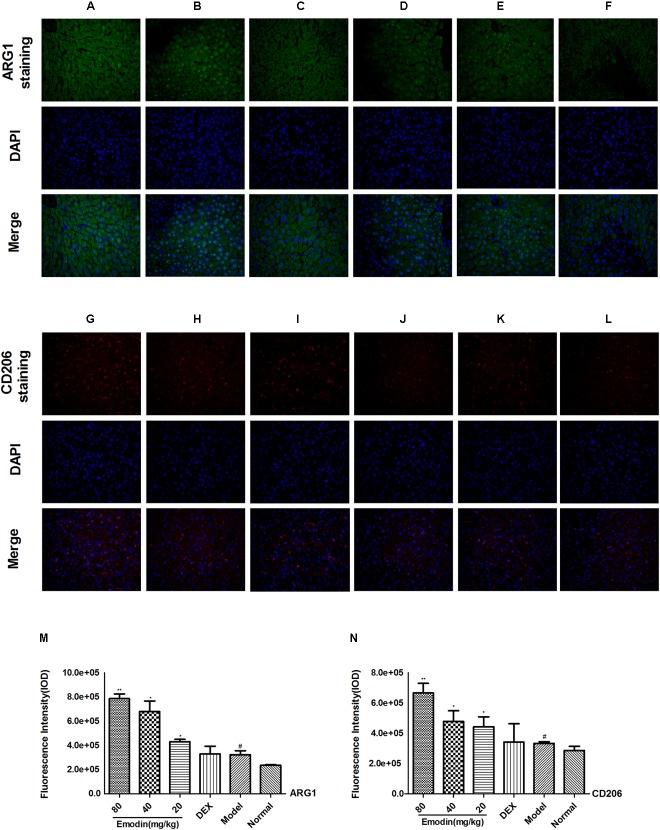
Effect of emodin on M2 macrophages in the liver by IF. The expression of mannose receptor ARG1 (green) and mannose receptor CD206 (red). Nuclei were visualized by DAPI (blue) staining. **(A/G)** Emodin (80 mg/kg) group, **(B/H)** emodin (40 mg/kg) group, **(C/I)** emodin (20 mg/kg) group, **(D/J)** DEX group, **(E/K)** model group, and **(F/L)** normal group (ARG1: **A–F,M**, CD206: **G–L,N**) figures were captured at 400X magnification. Data are shown as the mean ± SD. ^#^*P* < 0.05 the model group vs the normal group. ^∗^*P* < 0.05, ^∗∗^*P* < 0.01 vs model group.

ARG1 and CD206 are ideal phenotypic markers to identify M2 macrophages and can effectively distinguish M2 from M1 macrophage subtypes. We used the two indictors to analyze the expression of M2 macrophages in the liver with or without the treatment of emodin. As shown in **Figure 10**, the model group suffered severe tissue damage to inhibit the inflammatory response, and the M2 macrophage phenotype was activated in the livers of LPS-induced model mice. The levels of the M2 macrophage genes ARG1 and CD206 were increased in the model group compared with those in the normal group. Emodin had a remarkable effect on reducing liver injury and promoted anti-inflammatory M2 macrophage activation. To inhibit the inflammatory response, the M2 macrophage phenotype was activated in the liver of LPS-induced model mice. Compared with the model group, ARG1 and CD206 were significantly increased in the emodin groups under LPS stimulation (*P* < 0.05 or *P* < 0.01). The DEX group had no remarkable changes compared with the model group (**Figures 10A–N**; *P* > 0.05).

## Discussion

The hepatic inflammatory response, oxidative stress, macrophage activation (Ambade et al., 2012), and tissue hypoperfusion are primarily implicated in the pathogenesis of LPS-induced ALI (Zhang et al., 2014; Perea et al., 2017). Located in the hepatic sinus cavity, hepatic sinusoidal cells, liver macrophages, and leukocytes, which are important defensive cells that eliminate bacteria and toxins and antagonize infection, have the function of handling and delivering antigens as well as regulating the immune response in the body (Smedsrod et al., 2009). Leaking of aspartate AST and ALT is a characteristic of the injury and indicates acute degrees of hepatocyte necrosis and severe mitochondrial dysfunction and damage (La Mura et al., 2013; Jia et al., 2014). In this study, we examined the possible molecular mechanisms and protective effects of emodin on LPS-induced ALI *in vitro* and *in vivo*. The results of this study clearly indicated that emodin could significantly inhibit the expression levels of the MyD88 and TRIF signaling pathways on the TLR4 pathway and reduce the expression levels of TNF-α and IL-6. Eventually, the administration of emodin markedly suppressed the inflammatory response and subsequent liver injury.

Because the overproduction of inflammation has been implicated in the development and maintenance of ALI, early potent interventions to control liver injury development are particularly important. The pharmacological effects of DEX mainly concentrated on anti-inflammatory (Tolaj et al., 2017), immunosuppressive (Wang et al., 2013), anti-endotoxic (Zhang et al., 2017), and anti-shock (Yang et al., 2010) effects. While researchers have demonstrated that DEX has shown the ability to enhance the resistance of organ injury and improve the outcome of infection, its limitation is that it cannot be used for a long time (Kengatharan et al., 1996; Wang et al., 2013; Sun et al., 2017). Long-term use of glucocorticoids can cause many adverse effects, including weight gain, hyperglycemia, osteoporosis, cataracts, and increased risk of opportunistic infections. Thereby, it is very important to find new drugs with reliably effective treatments and fewer side effects to LPS-induced liver injury.

Previous studies have found that the major pharmacological activities of emodin are focused on anti-inflammatory, anti-tumor, anti-fibrosis, and hepatoprotective activities (Dong et al., 2009; Ding et al., 2016). For example, emodin suppresses the LPS-induced expression levels of MCP-1 and E-selectin, as well as pulmonary inflammation or edema (Xiao et al., 2014). Emodin weakens ethanol-induced liver damage and attenuates EtOH-induced toxicity in HepG2/CYP2E1 cells (Qian et al., 2011). Emodin showed the capability to prevent the oxidative liver damage induced by CCl4 (Lee et al., 2012). A previous study found that emodin alleviates intrahepatic cholestasis in young rats by regulation of the liver farnesoid X receptor (FXR) pathway (Ding et al., 2016). This study was performed to explore the molecular mechanisms and protective effects of emodin via the attenuation of TLR4 signaling pathways in macrophages and in mice (Chen et al., 2017). TLRs activation, specifically the TLR4 signal pathway in response to its activation by bacterial LPS-TLR4, has been considered a main component of the liver inflammatory response (Singh et al., 2017). The TLR4 ligand, LPS, interacts with TLR4 by binding to a hydrophobic pocket in MD2 (Koo et al., 2013). TLR4 activation leads to responses of MyD88-dependent and TRIF-dependent intracellular signaling pathways (Kaczorowski et al., 2009; Harlow et al., 2012) and provokes the MyD88 and TRIF pathways to trigger a series of inflammatory responses. Previous studies have shown that TLR4 recruits MyD88 and accepts TRAF6, using myelogenous differentiation factor 88-adapter like protein (MAL) as a bridging adaptor, which in turn activates NF-κB inducible kinase, phosphorylates I-κB kinase, and releases NF-κB from the IκB/NF-κB compound migrating into the nucleus, producing a series of inflammatory factors (Jiang et al., 2005; Xu et al., 2017). Another TLR4 signaling pathway is involved via a TRIF-related adaptor molecule (TRAM)-Trif-dependent mechanism by binding to activated TANK-binding kinase 1 (TBK1). These kinases eventually activate transcription factors such as IRF-3 and NF-κB (Iracheta-Vellve et al., 2016). Thus, the TLR4 signaling pathway, a potentially important therapeutic target, has a prominent role in LPS-associated ALI (Li et al., 2017).

In our research, we provide evidence that emodin was able to suppress inflammatory responses in both *in vitro* and *in vivo* experiments. For the in *vitro* experiment, the TLR4 signaling pathway was activated by LPS. Compared with the model group, the mRNA and protein expression levels of TLR4, MyD88, TIRAP, IRF-5, TRAF-6, TRIF, IRF-3, AP-1, and NF-κB were significantly inhibited in the emodin groups. Then, we used synthetic TLR4-siRNA to knockdown TLR4 expression. When TLR4 was knocked down and when LPS activated RAW 264.7 cells, the mRNA and protein expression levels of TLR4, MyD88, TIRAP, IRF-5, TRAF-6, TRIF, IRF-3, AP-1, and NF-κB were also inhibited by emodin. In addition, to confirm the efficacy of emodin, we treated RAW 264.7 cells with lentivirus vectors to overexpress TLR4. When TLR4 was excessively expressed and when LPS activated cells, the results showed that emodin had an obvious effect of inhibiting TLR4 and its downstream mediators followed by LPS, even if TLR4 was downregulated or overexpressed.

In the animal experiment, the results suggested that emodin protected against liver injury induced in mice by LPS through the inhibition of the inflammatory response via TLR4. Our findings are in accordance with other studies in which the mRNA expression of TLR4 increased significantly in severe hepatic damage (Ben et al., 2012). In the animal model, emodin also suppressed mRNA and protein levels of TLR4 and inhibited the downstream molecular levels of MyD88, TIRAP, IRF-5, TRAF-6, TRIF, IRF-3, AP-1, and NF-κB. In the liver, macrophages recognize pathogenic bacteria through TLR on their surface when tissue injury or pathogenic bacteria invade, differentiating into M1 macrophages, which participate in inflammatory reactions. To counteract liver tissue to the inflammatory response, macrophages are polarized M1 into M2 macrophages. The anti-inflammatory macrophage M2 phenotype depresses the proinflammatory response, while promoting tissue repair and restoration of homeostasis (Zhang et al., 2008; Wynn et al., 2013). Emodin has anti-inflammatory and antioxidation effects, reduces liver cell damage, attenuates liver function impairment, and has a significant protective effect on ALI (Jia et al., 2014; Ding et al., 2016). M2 macrophages play important roles in tissue repair and inflammation resolution and are a characteristic of high expression of Mrc1, ARG1, YM1, and so on (Iwanowycz et al., 2016). Therefore, we found that emodin increased M2 macrophage activation and promoted ARG1 and CD206 production in a dose-dependent fashion. As the model group suffered severe tissue damage, to counteract the tissue damage, the markers of ARG1 and CD206 onto M2 macrophages increased significantly. After establishing the model and providing treatments, we monitored the pathological changes in the liver as well as ALT and AST levels in serum and evaluated the tissue protective effects of emodin. As expected, emodin exerted liver-protective effects by attenuating the pathological damage and reducing serum AST and ALT levels and decreased the production of inflammatory mediators such as TNF-α and IL-6 in the LPS-induced ALI model in mice.

Our study has some limitations. In our experiments, liver injury in an animal model is a process with rapid onset. Thus, to better understand and characterize LPS-induced ALI, follow-up long-term studies are required to further assess the clinical benefits.

## Conclusion

Discovering effective, safe, and efficient treatments for the prevention and therapy of LPS-induced ALI is a major challenge. This study demonstrated that emodin treatment not only effectively reduced TLR4 and its downstream molecules but also suppressed the expression of TNF-α and IL-6 protein in LPS-induced ALI *in vitro* and *in vivo* and increased M2 macrophage activation, which reduces inflammation and improves liver injury. These results indicate that emodin may be a potential therapeutic agent for the prevention and treatment of ALI. The clinical relevance of these experimental findings should be evaluated in future studies.

## Ethics Statement

All experiments and animal care abided by internationally accepted principles and the Guidelines for the Care and Use of Laboratory Animals of Huazhong University of Science and Technology and were approved by the Ethics Committee of Union Hospital, Tongji Medical College, Huazhong University of Science and Technology.

## Author Contributions

LZ supervised the entire study. YD and PL designed and performed all experiments in cultured cells and *in vivo*. Z-LC, S-JZ, and Y-QW performed the animal feeding and evaluation of animal model histopathology. LL, XZ, and XC performed the data analysis. PL wrote the manuscript. All authors discussed the results and commented on the manuscript.

## Conflict of Interest Statement

The authors declare that the research was conducted in the absence of any commercial or financial relationships that could be construed as a potential conflict of interest.
